# Aβ ‐induced excessive mitochondrial fission drives type H blood vessels injury to aggravate bone loss in APP/PS1 mice with Alzheimer's diseases

**DOI:** 10.1111/acel.14374

**Published:** 2024-10-16

**Authors:** Weidong Zhang, Fan Ding, Xing Rong, Qinghua Ren, Tomoka Hasegawa, Hongrui Liu, Minqi Li

**Affiliations:** ^1^ Department of Bone Metabolism School and Hospital of Stomatology, Cheeloo College of Medicine, Shandong University & Shandong key Laboratory of Oral Tissue Regeneration & Shandong Engineering Research Center of Dental Materials and Oral Tissue Regeneration & Shandong Provincial Clinical Research Center for Oral Diseases Jinan China; ^2^ Center of Osteoporosis and Bone Mineral Research Shandong University Jinan China; ^3^ Developmental Biology of Hard Tissue, Graduate School of Dental Medicine, Faculty of Dental Medicine Hokkaido University Sapporo Japan; ^4^ School of Clinical Medicine, Jining Medical University Jining China

**Keywords:** Alzheimer's disease, bone loss, GSK‐3β, mitochondrial fission, type H blood vessels

## Abstract

Alzheimer's diseases (AD) patients suffer from more serious bone loss than cognitively normal subjects at the same age. Type H blood vessels were tightly associated with bone homeostasis. However, few studies have concentrated on bone vascular alteration and its role in AD‐related bone loss. In this study, APP/PS1 mice (4‐ and 8‐month‐old) and age‐matched wild‐type mice were used to assess the bone vascular alteration and its role in AD‐related bone loss. Transmission electron microscopy, immunofluorescence staining and iGPS 1.0 software database were utilized to investigate the molecular mechanism. Mitochondrial division inhibitor (Mdivi‐1) and GSK‐3β inhibitor (LiCl) were used to rescue type H blood vessels injury and verify the molecular mechanism. Our results revealed that APP/PS1 mice exhibited more serious bone blood vessels injury and bone loss during ageing. The bone blood vessel injury, especially in type H blood vessels, was accompanied by impaired vascularized osteogenesis in APP/PS1 mice. Further exploration indicated that beta‐amyloid (Aβ) promoted the apoptosis of vascular endothelial cells (ECs) and resulted in type H blood vessels injury. Mechanistically, Aβ‐induced excessive mitochondrial fission was found to be essential for the apoptosis of ECs. GSK‐3β was identified as a key regulatory target of Aβ‐induced excessive mitochondrial fission and bone loss. The findings delineated that Aβ‐induced excessive mitochondrial fission drives type H blood vessels injury, leading to aggravate bone loss in APP/PS1 mice and GSK‐3β inhibitor emerges as a potential therapeutic strategy.

## Graphical abstract

Peripheral circulatory Aβ aggravates the apoptosis of type H ECs, leading to impaired vascularized osteogenesis in AD mice. Further mechanism exploration found Aβ promotes the transfer of Drp1 from the cytosol to the mitochondria by activating GSK‐3β to phosphorylate Drp1 at ser616, leading to excessive mitochondrial fission. Abundant damaged mitochondria appeared and promoted the apoptosis of Type H ECs, due to Aβ‐induced excessive mitochondrial fission.

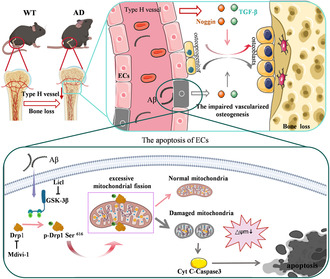

**Abbreviations**
AD: Alzheimer's diseases
Aβ: β‐amyloid
Drp1: dynamin‐related protein 1
ECs: endothelial cells
EMCN: endomucin
GSK‐3β: glycogen synthase kinase‐3beta

## 1 INTRODUCTION

Alzheimer's disease (AD) is a progressive age‐related neurodegenerative disease worldwide, with the impaired memory and cognition (Chhetri et al., 2019). The pathological feature of AD is characterised by the emergence of β‐amyloid (Aβ) plaques and neurofibrillary tangles in brain, resulting in neuronal injury. The preferentially impaired neurons are tightly associated with AD patients' memory and cognitive abilities, leading to dementia that is the hallmark of AD (Beata et al., 2023). While AD is notorious for the impairment of memory and cognition, AD patients also exhibit higher incidence of bone loss and skeletal fractures compared with cognitively normal individuals of similar age, significantly enhancing the healthcare burden on their families and caregivers (AD facts and figures 2023; Jiang et al., 2022; Liang & Wang, 2017). However, current understanding on the pathological relationship between AD and bone loss remains elusive. A better understanding of the pathological mechanism underlying the bone loss among AD patients may provide a novel insight for development of therapeutic strategies.

It was reported that age‐related vascular alteration accompanies or even precedes the development of AD, emerging as a predictor of AD (Cortes‐Canteli & Iadecola, 2020). Prior research demonstrated that the accumulation of Aβ is a likely contributor of vascular alteration (Koemans et al., 2023). The alteration includes but not limited to intracranial atherosclerosis, reduced cerebral microvascular density and cerebral amyloid angiopathy, playing a notable role in AD patients' memory and cognitive abilities (Ting et al., 2023). Of note, the vascular alteration is not only limited to cerebral vessels, but also is associated with peripheral vessels, providing the ‘pathological bridge’ between AD and other systemic diseases, such as large artery stiffening and retinal vascular disease (Nordestgaard etal., 2022; Shi et al., 2020). Bone, as an organ rich in blood vessels, is indispensable for maintaining bone health. Apart from facilitating blood supply, bone blood vessels also serve as niches for stem cells, playing a pivotal role in regulating bone formation (Tuckermann & Adams, 2021). Therefore, the bone vascular alteration is associated with bone loss. However, few studies have concentrated on addressing the alteration of bone blood vessels and its role in bone loss among AD patients.

Recently, a subtype of blood vessels was found to be tightly associated with osteogenesis, characterized by high expression level of CD31 and Endomucin (CD31^hi^Emcn^hi^), and it was termed type H blood vessels (Peng et al., 2020). Human type H blood vessels were confirmed to be a sensitive biomarker of bone mass. The reduction of type H blood vessels has been identified as a critical contributor of impaired bone microarchitecture in aging and postmenopausal osteoporosis (Wang et al., 2017). Type H blood vessels injury could be a critical reason for poor bone formation and bone loss among AD patients, while the underlying mechanism remains elusive. It has been proposed that the osteogenic effect of type H blood vessels could result from the communication between vascular endothelial cells (ECs) and osteoblast lineage (Liu et al., 2023). ECs in type H blood vessels play an active role in directing bone formation through the secretion of factors that support perivascular osterix^+^ osteoprogenitors in the bone marrow (Ramasamy et al., 2014). The spatial and temporal link between angiogenesis and osteogenesis has been defined as ‘angiogenic–osteogenic coupling’ (Kusumbe etal., 2014). Recent study identified that 40%–60% of brain‐derived Aβ is cleared away in peripheral organs, such as liver (Cheng et al., 2023). Peripheral circulatory Aβ could aggravate the damage of ECs in type H blood vessels and result in uncoupled angiogenesis and osteogenesis. However, the underlying pathophysiological mechanisms need to be clarified.

Mitochondria are identified to be dynamic organelles which continually experience fusion and fission by altering their number and shape to satisfy to the need of cell survival (Li & Liu, 2018). Recent studies reported that dysregulation of mitochondrial fission leads to cell apoptosis through generation of excessive mitochondrial fragmentation and abnormal energy metabolism (Ansari et al., 2022; Yi et al., 2022). Drp1, a member of the dynamin family of GTP‐binding proteins, is the primary mediator of mitochondrial fission (Rahmani et al., 2023). Various posttranslational modifications (PTMs) of Drp1 were reported to serve a key role in mitochondrial fission (Jin et al., 2021). Phosphorylation is considered as the best characterized PTMs of Drp1, which can play dual effects depending on the specific site modified. Previous evidence identified that the activity of glycogen synthase kinase‐3β (GSK‐3β), an important phosphorylated kinase, is abnormally elevated in AD patients (Cheng et al., 2024). Hence, the abnormal activation of GSK‐3β may be the main culprit for excessive mitochondrial fission and apoptosis of type H ECs.

In the present study, we sought to clarify the alteration of bone vascular and its role in AD‐related bone loss. We identified that Aβ could aggravate the impairment of bone blood vessels, especially type H blood vessels, leading to the impaired vascularized osteogenesis. Mechanistic dissection revealed that Aβ induced the apoptosis of ECs by promoting excessive mitochondrial fission. GSK‐3β activation is a critical contributor to excessive mitochondrial fission in ECs. These findings provide a novel prospect for bone loss complicated with AD.

## 2 METHODS

### 2.1 Animals handling and drug administration

Male APP/PS1 mice (4‐ or 8‐month‐old) and age‐matched wild‐type (WT) mice were obtained from the Huachuang Sino Co., Ltd. (Jiangsu, China). The Morris water maze (MWM) test was used to assess the ability of spatial learning and memory abilities of mice. Moreover, Mdivi‐1(MedChemExpress, HY‐15886, 50 mg/kg, twice/week) and LiCl (MedChemExpress, HY‐W094474, 30 mg/Kg, daily) were utilized to verify the role of mitochondrial fission in type H blood vessels injury in 8‐month APP/PS1 mice compared with placebo group. This process lasted for 4 weeks and the mice were anesthetized and sacrificed. Femur and tibia were preserved for subsequent experiments. The retro‐orbital blood sampling method was applied to collect the blood samples. Serum samples were separated by centrifugation at 3000 rpm for 10 min. Aβ content was detected in serum by ELISA (KYY‐44819 M2, Keyybio, Shandong, China) as following the instruction. This study was approved by the Animal Research and Ethics Review Committee of Stomatological Hospital of Shandong University (Jinan, China; Approval NO. 20230362).

### 2.2 Bone mass measurement

Following fixation, the dissected bone was scanned using high‐resolution micro‐computed tomography (μCT) (PerkinElmer, Quantum GX2). Analyze 12.0 (PerkinElmer, Quantum GX2) software was employed to reconstruct the three‐dimensional (3D) images and analyze parameters, including bone mineral density (BMD) and volume per tissue volume (BV/TV). Next, the tibia was decalcified in 0.5 M EDTA at 4°C for 21 days. The paraffin‐embedded tissue sections were obtained as described previously (Lu et al., 2022). Hematoxylin and eosin (HE) staining was performed to assess the bone mass on the basis of the method outlined in prior research (Lu et al., 2022).

### 2.3 Immunofluorescence (IF) and immunohistochemistry (IHC)

In addition to paraffin‐embedded sections, some tibias were utilized to prepare frozen slices. The decalcified bones were incubated in 20% sucrose for 24 h and then embedded into Tissue‐Tek O.C.T. Compound (4583; SAKURA, Tokyo, Japan). The embedded tissue sections were cut into 40‐μm thick slices. For IF staining, bone slices were permeabilized for 30 min in 1% Triton X‐100 and blocked with 1% bovine serum albumin‐phosphate‐buffered saline (BSA‐PBS) for 30 min. Subsequently, it was attempted to incubate tissue slices with the following primary antibodies diluted in 1% BSA‐PBS: anti‐CD31 (ab9498; Abcam, Cambridge, UK), anti‐endomucin (ab106100; Abcam), anti‐osterix (ab209484; Abcam) and anti‐Aβ (ab201060; Abcam) overnight at 4°C. On the second day, tissue slices were incubated with fluorescent secondary antibodies conjugated with Alexa 488, 594 or 647. Nuclei were stained with 40, 60‐diamidino‐2‐phenylindole (DAPI) (ab104139; Abcam). Following coverslipping, the sections were examined using a confocal laser microscope. Similarly, treated cells were permeabilized with 1% Triton X‐100 after fixation with 4% paraformaldehyde. After blocking in 1% BSA‐PBS, the cells were incubated overnight at 4°C with anti‐p‐Drp1 (AF8470; Affinity, Jiangsu, China) and anti‐GSK‐3β (67329‐1‐lg; Proteintech, Wuhan, China) antibodies. On the following day, the cells were treated with fluorescent secondary antibodies and DAPI. Subsequently, the cells were observed using a super‐resolution confocal microscope (DeltaVision OMX Flex; Leica, Wetzlar, Germany). The immunohistochemical staining procedure is essentially identical to IF, where paraffin‐embedded tissue sections were treated with anti‐ALP (ET1601‐21; HUABIO Co., Ltd., Shanghai, China) and anti‐RUNX2 (ab192256, Abcam) antibodies, and diaminobenzidine (Sigma‐Aldrich, MO, USA) was utilized for visualizing the immune response. Tissue slices were then retained with methyl green.

### 2.4 Flow cytometry and enzyme‐linked immunosorbent assay (ELISA)

For the analysis of CD31^hi^Emcn^hi^cells in bone, femurs of mice were collected after removing muscles and periosteum around bone. After crushing the metaphysis and diaphysis, bone marrow cavity was rinsed with 1 mL ice‐cold PBS. Following centrifugation, the supernatant was utilized for ELISA with Noggin (KYY‐0911 M2, Keyybio) and TGF‐β (KE10005, Proteintech), while the cell precipitate underwent treatment with collagenase (ST2303, 0.5 mg ml^−1^; Beyotime Institute of Biotechnology, Shanghai, China) to obtain single‐cell suspensions. These cells were subsequently subjected to red blood cell lysis using the procedure outlined by the red blood cell lysate (Solarbio, R1010). After filtration and washing, the cells were incubated with endomucin antibody (sc‐65,495, Santa) for 45 min at 4°C, followed by washing and subsequent incubation with fluorescent secondary antibodies conjugated with FITC. Thereafter, the cells were washed and further incubated with PE‐conjugated CD45 (E‐AB‐F1136D, 1:20; Elabscience, Wuhan, China) and APC‐conjugated CD31 (E‐AB‐F1180E, 1:20; Elabscience) antibodies for 45 min at 4°C. The NovoCyte Advanteon flow cytometer was utilized for data acquisition.

### 2.5 Isolation and culture of bone ECs

Similarly, metaphysis in tibia and femurs were collected and crushed. Collagenase and red blood cell lysis were applied to obtain single‐cell suspension. Bone ECs were sorted by Magnetic Activated Cell Sorting (MACS) using endomucin antibody (sc‐65,495, Santa) and Dynabeads coated with anti‐rat antibody (SA00003‐11, Proteintech). Sorted bone ECs were counted and cultured in endothelial cell growth medium (38,998, SclenCell). At first passage, cells were sorted again by MACS. Cells were fed every third day and applied for the following experiments.

### 2.6 Apoptosis analysis by flow cytometry and TUNEL staining and the assessment of pro‐osteogenic factors

ECs were seeded into 6‐well plates and treated with different concentrations of Aβ (MCE, HY‐P1388). 40 μM Z‐VAD‐FMK (APExBIO, A1902) was applied to inhibit the apoptosis of ECs. The apoptosis analysis was carried out as outlined in the manufacturer's instructions. In brief, the collected cells were incubated with binding buffer containing Annexin V‐FITC and PI (BD, 556547, 5 μL; BD Biosciences, Franklin Lakes, NJ, USA). Finally, the apoptosis of cells was assessed by a flow cytometer. One‐Step TUNEL Apoptosis Assay kit (C1086, Beyotime Institute of Biotechnology) was also utilized to assess cell apoptosis. The cells were pretreated with mdivi‐1 or LiCl, and subsequently treated with 5 μM Aβ. Thereafter, the treated cells were fixed with 4% paraformaldehyde and permeated with 1% Triton X‐100. Next, the cells were exposed to the TUNEL reaction mixture and phalloidin in accordance with the manufacturer's instructions. Additionally, nuclei were stained with DAPI. The number of TUNEL‐positive cells was counted using a fluorescence microscope. Apoptosis in tibia sections was detected using TUNEL staining. TUNEL‐ and CD31‐positive cells were considered as the apoptotic ECs. Similarly, the supernatant of treated cell was collected and detected by the ELISA kits (WELLBIO, EH10318s, EH10908s) to assess the pro‐osteogenic factors, such as TGF‐β and Noggin.

### 2.7 Alkaline phosphatase (ALP) staining and alizarin red (AR) staining

Murine osteoprogenitors were isolated from femurs of 4‐week‐old WT mice(Chen et al., 2022). Identification of osteoprogenitors was carried out using flow cytometry with CD45, CD73, CD90 and CD105 markers. After identification, the cells were divided into the different groups. As described in Figures 2c and 3e, the osteoprogenitors received mixed liquid involved osteogenic induction medium and supernatant from ECs at the ratio of 1:1. After induction of osteogenesis for 7 days, these cells were subjected to ALP staining according to the manufacturer's instructions. For AR staining, the cells were cultured in an osteogenic induction medium for 21 days. The images were captured using an optical microscope (CKX‐41; Olympus, Tokyo, Japan).

### 2.8 The analysis of mitochondrial morphology

The mitochondrial morphology in ECs was assessed through transmission electron microscopy (TEM). The treated cells were fixed with 2.5% glutaraldehyde. Subsequent procedures were conducted by an expert in the electron microscopy facility at Shandong University. TEM images were captured using a Thermo Fisher Talos F200C transmission electron microscope. Furthermore, treated cells were incubated in a confocal dish with α‐MEM containing 100 nmol/L Mito Tracker Green (C1048, Beyotime Institute of Biotechnology) and Hoechst living cell staining solution (C1027, Beyotime Institute of Biotechnology). The cells were observed under a super‐resolution confocal microscope (DeltaVision OMX Flex, Leica). The length of mitochondria was analyzed by ImageJ software.

### 2.9 The analyses of mitochondrial membrane potential (MMP)

MMP was measured using a JC‐1 assay kit (C2006, Beyotime Institute of Biotechnology) as described previously (Cui et al., 2023). Briefly, the treated cells were incubated with JC‐1 dye for 20 min and observed under a fluorescence microscope (DMi8, Leica). The images were obtained under a fluorescence microscope (DMi8, Leica). The fluorescence intensity was analyzed by ImageJ software.

### 2.10 Western blotting

As our previous methods (Gao et al., 2023), the total protein from tibia or treated cells was extracted using the RIRA kit (02408/60412; CwBio Biotechnology, Beijing, China). Additionally, mitochondrial fractions were isolated using a cell‐mitochondrial isolation kit (PK10016, Proteintech). Cytosolic protein was isolated from the treated cells using a cytoplasmic protein extraction kit (P0027, Beyotime Institute of Biotechnology). Protein concentration was detected using a BCA protein assay kit (P0009, Beyotime Institute of Biotechnology). SDS‐PAGE was applied to separate the equal amounts of protein from each group. Thereafter, the separated protein was transferred onto PVDF membranes. After blocking with BSA, the PVDF membranes were incubated with anti‐ALP (A0514, ABclonal), anti‐RUNX2 (D130‐3, MBL), anti‐Drp1 (12957‐1‐AP, Proteintech), anti‐caspase3 (ab13847, Abcam), anti‐Bax (50599‐2‐lg, Proteintech), anti‐Bcl2 (68103–1‐1 g, Proteintech), anti‐Cytochrome C (10993‐1‐AP, Proteintech), anti‐GAPDH (10494‐1‐AP, Proteintech), and anti‐COX IV (11242‐1‐AP, Proteintech) antibodies overnight at 4°C. After being washed, the membranes were incubated with a goat anti‐rabbit (or anti‐mouse) secondary antibody. Blots were displayed using enhanced chemiluminescent reagents (YA0372; Beijing Solarbio Science & Technology Co., Ltd., Beijing, China).

### 2.11 The prediction of Drp1 phosphorylated kinase

The iGPS 1.0 software was utilized to predict Drp1 phosphorylated kinase.

### 2.12 Docking analysis

The RCSB database and UniProt database were applied to obtain the structures of GSK‐3β and Drp1. They were subjected to energy minimization and molecular docking using MOE 2019.1. Pymol2.1 was applied to visualize figures.

### 2.13 Statistical analysis

The data were expressed as mean ± standard deviation based on at least three independent biological replicates. GraphPad Prism 6.0 software (GraphPad Software, Inc., San Diego, CA, USA) was utilized to carry out statistical analysis. While *t*‐test was employed to analyze the differences between two groups, one‐way or two‐way analysis of variance (ANOVA) was applied to assess the differences among three or more groups. Bonferroni post‐hoc test was used to analyze differences among multiple comparisons. *p* < 0.05 was considered statistically significant.

## 3 RESULTS

### 3.1 APP/PS1 mice exhibited more serious bone blood vessels injury and bone loss during ageing

**FIGURE 1:**
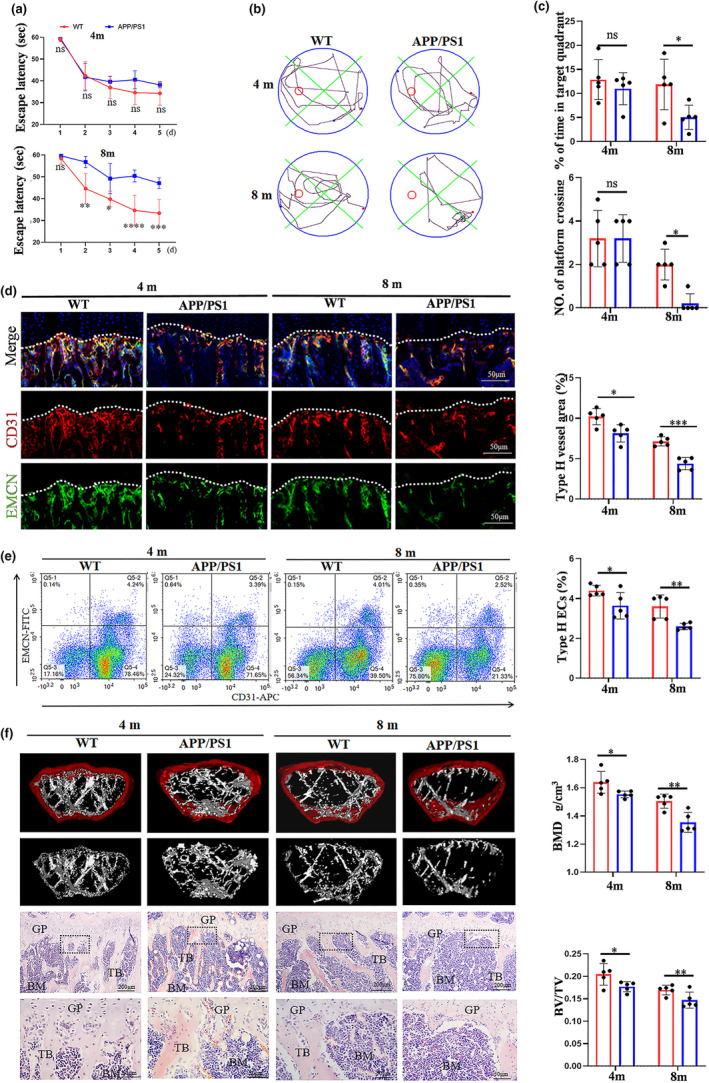
APP/PS1 mice exhibited more serious bone blood vessel injury and bone loss during ageing. (a) Escape latency of the MWM tests was assessed in APP/PS1 mice (4‐ or 8‐month‐old) and age‐matched wild‐type mice. (b) Representative traces of APP/PS1 mice and wild‐type mice in MWM test. (c) The target quadrant retention time (%). (d) Confocal image of EMCN^hi^ and CD31^hi^ (termed type H blood vessels) in APP/PS1 and wild‐type mice and histomorphometric quantitation of the area of type H blood vessels. Scale bar: 50 μm. (e) Representative flow cytometry plots of CD31^hi^Emcn^hi^ ECs (type H ECs) and the quantitation of type H ECs. (f) The assessment of bone mass by μCT and HE staining and quantitative parameters. Scale bar: 200, 50 μm. BMD, Bone mineral density, BV/TV, Bone Volume per Trabecular Volume. Values are presented as mean ± SEM. Two‐way ANOVA with Bonferroni's multiple comparisons test was performed in a. T‐ test was performed in c, d, e, f. (****p < 0.0001, ***p < 0.001, **p < 0.01, *p < 0.05).

To assess the alteration of bone vessels and the role of bone vessels injury in AD‐related bone loss, APP/PS1 mice and WT mice at 4 and 8 months of age were evaluated. The MWM test was initially conducted to evaluate the spatial memory deficits of APP/PS1 mice and WT mice. As illustrated in Figure 1a, there was no significant difference in the escape latency of APP/PS1 mice and WT mice at 4 months of age. Similarly, the results of probe test also exhibited no significant discrepancy (Figure 1b,c). However, the long escape latency was noted in 8‐month‐old APP/PS1 mice to find the platform compared with WT mice (Figure 1a). Meanwhile, 8‐month‐old APP/PS1 mice exhibited less number and time spent on the target zone relative to WT mice (Figure 1b,c). These results suggested that 8‐month‐old APP/PS1 mice had spatial memory deficits, rather than 4‐month‐old APP/PS1 mice. Furthermore, bone blood vessels were detected by IF staining of the tibial sections. It was found that the number of Emcn^hi^CD31^hi^ type H blood vessels adjacent to the growth plate was reduced in 4‐ and 8‐month‐old APP/PS1 mice compared with age‐matched WT mice (Figure 1d). Flow cytometry results further revealed a reduced presence of type H ECs in the femur of APP/PS1 mice at both 4 and 8 months of age compared with WT mice of corresponding ages (Figure 1e). The results of μCT indicated that 4‐month‐old APP/PS1 mice slightly presented bone loss (Figure 1f). The significant difference in bone loss was found in 8‐month‐old APP/PS1 mice and WT mice. Histologically, there were seemed to be decreased metaphyseal trabeculae in APP/PS1 mice, compared with WT mice, which were consistent with the results of μCT (Figure 1f). Notably, APP/PS1 mice exhibited more serious bone vessel injury accompanied by bone loss during ageing.

### 3.2 Bone blood vessels injury was accompanied by impaired vascularized osteogenesis in APP/PS1 mice

**FIGURE 2:**
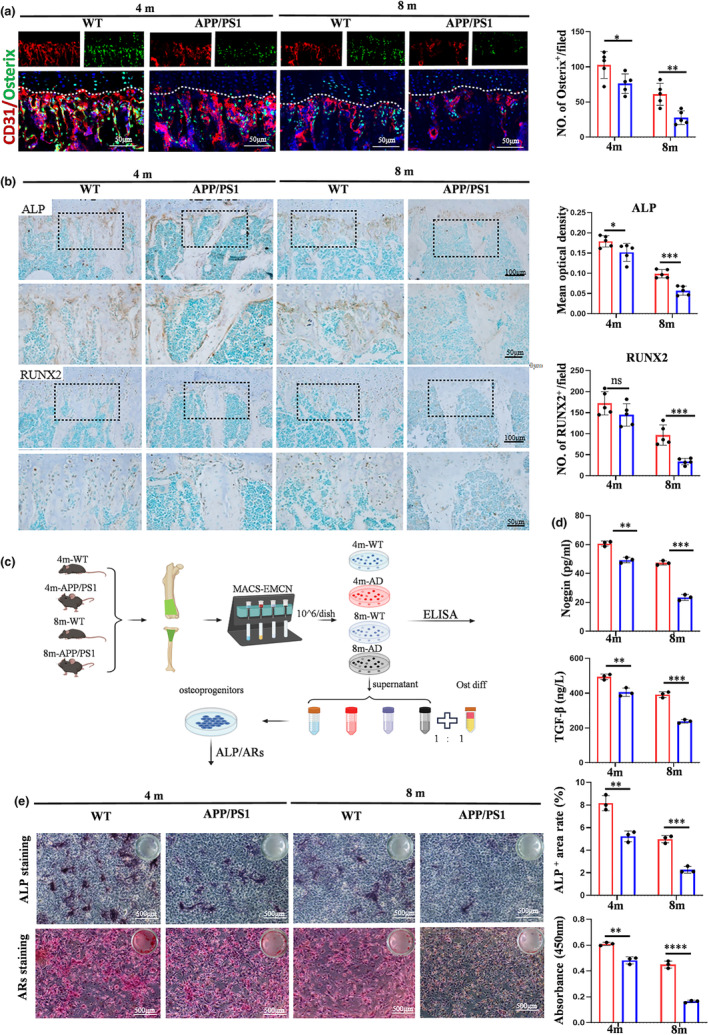
Bone blood vessels injury was accompanied by impaired vascularized osteogenesis in APP/PS1 mice. (a) Confocal image of Osterix^+^ (green) osteoprogenitors around CD31^hi^ vessels (red) and the quantitative analysis. Scale bar: 50 μm. (b) IHC analysis of ALP and RUNX_2_ in tibia of APP/PS1 and wild‐type mice. Scale bar: 100 μm, 50 μm. (c) Bone ECs were isolated from femoral and tibial metaphyseal region by Magnetic Activated Cell Sorting (MACS) using endomucin antibody. Bone ECs were subjected to ELISA. Osteogenic induction fluid mixed with supernatant collected from bone ECs. The mixed liquid was applied to culture the osteoprogenitors. (d) The levels of pro‐osteogenic factors were assessed by ELISA. (e) ALP staining and ARS staining. Values are presented as mean ± SEM. T‐ test was performed in a, b, d, e. (****p < 0.0001, ***p < 0.001, **p < 0.01, *p < 0.05).

As angiogenesis is tightly coupled with osteogenesis in bone, we investigated the alteration of vascularized osteogenesis in APP/PS1 mice. Histomorphometry observation found a significant decrease of Osterix^+^ osteoprogenitors around the type H blood vessels in APP/PS1 mice compared with age‐matched WT mice (Figure 2a). It suggested that there may be the impaired vascularized osteogenesis in APP/PS1 mice. Meanwhile, we investigated the activity of osteoblasts to reflect the bone formation rates. The weaker ALP immunoreactivity and fewer number of RUNX_2_^+^ osteoblasts were observed in the metaphysis of APP/PS1 mice by IHC staining (Figure 2b). Similarly, western blotting results also revealed lower expression levels, suggesting lower bone formation rates in APP/PS1 mice (Figure S1b). It was well known that type H blood vessels modulate osteogenesis by the interaction between vascular cells and osteoblastic cell lineage via secreting some pro‐osteogenic factors, such as noggin and TGF‐β. The reduced expression of noggin and TGF‐β in bone marrow of APP/PS1 mice revealed the impaired vascularized osteogenesis in APP/PS1 mice (Figure S2b). In order to further identify the impaired vascularized osteogenesis, bone special ECs were sorted from APP/PS1 mice and age‐matched WT mice (Figure 2c); (Figure S1c). The result of ELISA showed the reduced expression of pro‐osteogenic factors in freshly sorted bone ECs from APP/PS1 mice compared with age‐matched WT mice, especially 8‐month‐old APP/PS1 mice (Figure 2d). Meanwhile, supernatant collected from fresh bone ECs was applied to induce osteoprogenitors (Figure 2c). ALP staining and ARs staining demonstrated the decreased osteogenic ability of the supernatant from APP/PS1 mice compared with WT mice, especially 8‐month‐old APP/PS1 mice (Figure 2e). In a word, these results demonstrated that bone blood vessels injury was accompanied by impaired vascularized osteogenesis in APP/PS1 mice.

### 3.3 Aβ induced the impairment of vascularized osteogenesis by promoting the apoptosis of ECs

**FIGURE 3:**
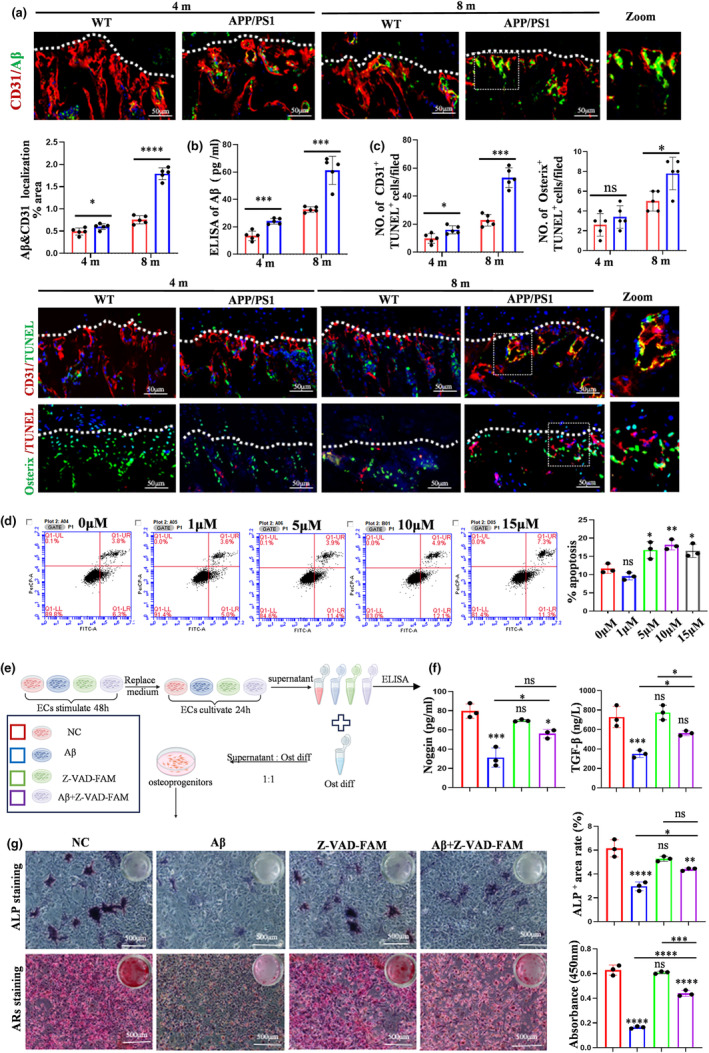
Aβ induced the impairment of vascularized osteogenesis by promoting the apoptosis of ECs. (a) Confocal images show the expression of Aβ in blood vessels. Scale bar: 50 μm. (b) The ELISA test of Aβ in serum. (c) TUNEL staining shows the apoptosis of ECs (CD31^+^TUNEL^+^) and osteoprogenitors (osterix^+^TUNEL^+^). Scale bar: 50 μm. (d) Flow cytometry analysis of apoptotic rates in ECs. (e) Schematic representing the role of Aβ‐induced apoptosis of ECs on the osteogenic difference of osteoprogenitors. (f) The ELISA test of Noggin and TGF‐β. (g) ALP staining and ARS staining. Values are presented as mean ± SEM. T‐test was performed in a, b, c. One‐way ANOVA with Bonferroni's multiple comparisons test was performed in d, f, g. (****p < 0.0001, ***p < 0.001, **p < 0.01, *p < 0.05).

Previous studies revealed that Aβ promotes the apoptosis of cerebral endothelial cell and serves a key role in aging‐related vascular pathologies (Carey etal., 2024; Khan & Qiu, 2023). Hence, we speculated that the bone blood vessels injury may be associated with Aβ. To examine this hypothesis, Aβ content in bone blood vessels was measured. The results of IF staining showed the increased Aβ deposition in bone blood vessels of 8‐month‐old APP/PS1 mice compared with that in WT mice (Figure 3a). Similarly, 4‐month‐old APP/PS1 mice also exhibited relative more deposition of Aβ than WT mice of the same age (Figure 3a). The ELISA of Aβ also exhibited the same trend in serum (Figure 3b). As described above, vascular ECs and osteoprogenitors serve a key role in the coupling of angiogenesis and osteogenesis. Therefore, it is logical to speculate that the impairment of vascularized osteogenesis was caused by Aβ‐induced injury of vascular ECs and osteoprogenitors. Indeed, co‐staining of frozen section of tibia with CD31 (ECs) and TUNEL found that the number of CD31^+^ TUNEL^+^ cells significantly increased in APP/PS1 mice, especially in 8‐month‐old mice (Figure 3c). Meanwhile, compared with WT group at the same age, APP/PS1 group also presented relatively more Osterix^+^ TUNEL^+^ cells. However, the number of apoptotic ECs was significantly increased greater than that of apoptotic osteoprogenitors during aging, suggesting ECs may be more susceptible to Aβ (Figure 3c). Considering ECs are more severely damaged in vivo observation, we further confirmed the effect of Aβ on ECs in vitro. ECs were treated with gradient concentration of Aβ and flow cytometry was subsequently employed to detect the apoptosis status of those treated ECs. The results revealed that low‐dose (1 μΜ) Aβ has a protective effect on cell survival. Whereas 5 μM, 10 μM, 15 μM Aβ significantly increased the apoptosis of ECs (Figure 3d). Next, pretreated with 40 μM Z‐VAD‐FMK significantly inhibited the apoptotic effect of Aβ on ECs (Figure S3a). These results demonstrated that Aβ promotes the apoptosis of ECs. In order to further clarify the relationship of Aβ and the impairment of vascularized osteogenesis, indirect co‐culture method was applied in the next step (Figure 3e). The pro‐osteogenic factors were decreased in Aβ group, while pre‐treated with Z‐VAD‐FMK alleviated the effect of Aβ on the expression of pro‐osteogenic factors, detected by ELISA (Figure 3f). Moreover, ALP and ARS staining results indicated that supernatant from Aβ‐treated ECs inhibited the osteogenic difference of osteoprogenitors compared with the NC group (Figure 3g). Altogether, these findings suggested that Aβ induced the impairment of vascularized osteogenesis by promoting the apoptosis of ECs.

### 3.4 Aβ‐induced excessive mitochondrial fission was required for the apoptosis of ECs

**FIGURE 4:**
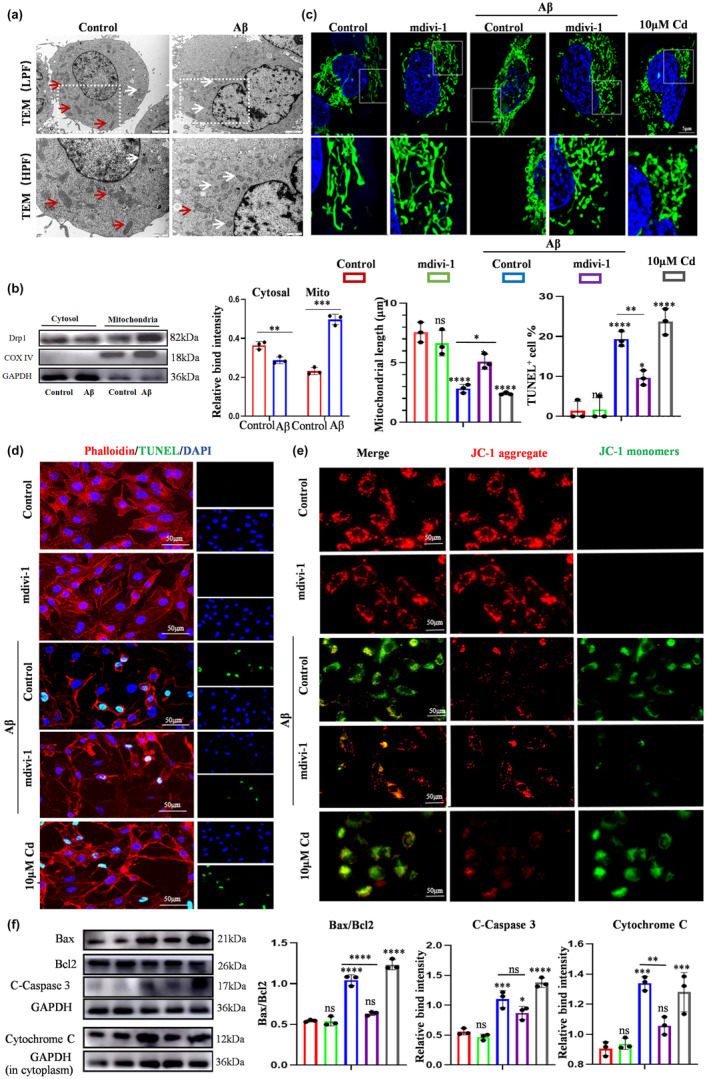
Aβ‐induced excessive mitochondrial fission was required for the apoptosis of ECs. (a) Transmission electron microscope observation of subcellular structure of ECs after treated with Aβ. Scale bar: 2 μm, 1 μm. White arrow, small punctate and rounded mitochondria; red arrow, filamentous or tubular mitochondria. (b) Western blot analysis of the expression of Drp1 in cytoplasm and mitochondria with or without the treatment of Aβ. (c) The Mito Tracker staining showed the role of Aβ on mitochondrial fission. (d) TUNEL staining and phalloidin staining to assess the role of Aβ‐induced excessive mitochondrial fission in the apoptosis of ECs. (e) The mitochondrial membrane potential was measured by JC‐1 dye. (f) Western blot analysis of the expression of apoptosis‐related proteins. Values are presented as mean ± SEM. T‐ test was performed in b. One‐way ANOVA with Bonferroni's multiple comparisons test was performed in c, d, e, f. (****p < 0.0001, ***p < 0.001, **p < 0.01, *p < 0.05).

To further explore the mechanism of ECs' apoptosis, subcellular structure of ECs was observed after treatment with 5 μM Aβ (Figure 4a). Ultrastructural analysis using TME revealed that ECs treated with Aβ displayed a higher quantity of small punctate and rounded mitochondria with aberrant morphology compared with the control group, suggesting that mitochondrial fragmentation and damage could potentially contribute to EC apoptosis. Studies have demonstrated that mitochondrial fragmentation may result from excessive mitochondrial fission (Sheridan & Martin, 2010). As the critical mediator of mitochondrial fission, Dynamin‐related protein 1 (Drp1) expression level ascertained that Aβ exerted an accelerative effect on mitochondrial fission by promoting the translocation of Drp1 from cytoplasm to mitochondria (Figure 4b). To further confirm this result, a mitochondrial fission inhibitor, mdivi‐1, was recruited to restrain Aβ‐induced excessive mitochondrial fission. The shortened and less interconnected mitochondrial structures found in the Aβ group were significantly improved to a filamentous or tubular pattern following pretreatment with mdivi‐1, as visualized by mitochondrial fluorescent probes (Figure 4c). To clarify the relationship between Aβ‐induced excessive mitochondrial fission and apoptosis of ECs, TUNEL and phalloidin staining was used to assess the apoptosis status of ECs. The result showed that mdivi‐1 rescued Aβ‐induced apoptosis of ECs, suggesting Aβ‐induced excessive mitochondrial fission induced the apoptosis of ECs (Figure 4d). Moreover, the results of analysis of MMP also demonstrated that Aβ‐induced excessive mitochondrial fission induced the mitochondrial damage (Figure 4e). The release of cytochrome C and caspase activation indicated that inhibition of mitochondrial fission could rescue Aβ‐induced apoptosis of ECs, observed by western blot (Figure 4f). Therefore, these results demonstrated that Aβ‐induced excessive mitochondrial fission was required for the apoptosis of ECs.

### 3.5 Mdivi‐1 effectively attenuated bone blood vessels injury and bone formation in APP/PS1 mice

**FIGURE 5:**
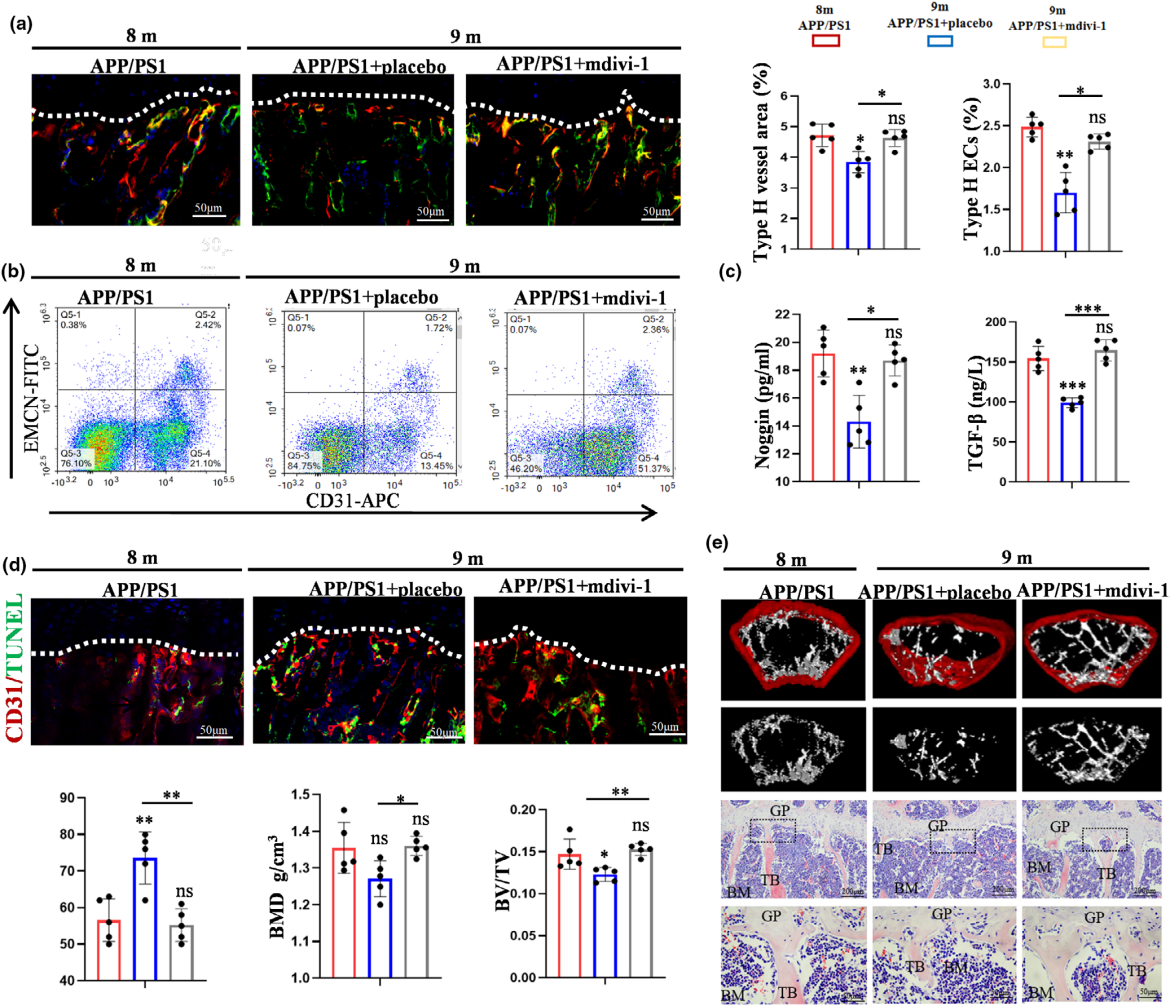
Mdivi‐1 effectively attenuated bone blood vessels injury and bone formation in APP/PS1 mice. (a) The application of mdivi‐1 protects the type H blood vessels injury. Scale bar: 50 μm. (b) Representative flow cytometry plots of CD31^hi^Emcn^hi^ ECs (type H ECs) represents the role of mdivi‐1. (c) Noggin and TGF‐β were measured by ELISA. (d) TUNEL staining presents the protective of mdivi‐1 on ECs in APP/PS1 mice. Scale bar: 50 μm. (e) The assessment of bone mass by μCT and HE staining and quantitative parameters. Scale bar: 200, 50 μm. BMD: Bone mineral density. Values are presented as mean ± SEM. One‐way ANOVA with Bonferroni's multiple comparisons test was performed in a, b, c, d, e. (***p < 0.001, **p < 0.01, *p < 0.05).

To explore the role of Aβ‐induced excessive mitochondrial fission in the type H blood vessel injury, mdivi‐1 was injected into 8‐month‐old APP/PS1 mice versus the placebo group. IF staining revealed that mdivi‐1 group mice exhibited more type H blood vessels than that in placebo group (Figure 5a). Similarly, compared with that in placebo group, the decrease in the number of ECs showed a slowing trend in mdivi‐1 group, as detected by flow cytometry (Figure 5b). IF and flow cytometry results demonstrated that mdivi‐1 ameliorated the bone blood vessel injury during ageing in APP/PS1 mice. ELISA results also identified that mdivi‐1 promoted the expression levels of the pro‐osteogenic factors in bone marrow, which further clarify the role of Aβ‐induced excessive mitochondrial fission in vascularized osteogenesis (Figure 5c). Moreover, TUNEL staining was employed to assess the effect of mdivi‐1 on the apoptosis of ECs. The decreased CD31^+^ TUNEL^+^ cells confirmed that mdivi‐1 exerted a protective role in type H ECs of APP/PS1 mice (Figure 5d). Finally, the assessment of bone mass by μCT and HE provided more robust evidence, in which Aβ‐induced excessive mitochondrial fission was tightly associated with the type H bone blood vessel injury, resulting in bone loss (Figure 5e). These results suggested that mdivi‐1 effectively attenuated bone blood vessels injury and bone formation in APP/PS1 mice.

### 3.6 GSK‐3β was found as a key regulatory target of Aβ‐induced excessive mitochondrial fission and bone loss

**FIGURE 6:**
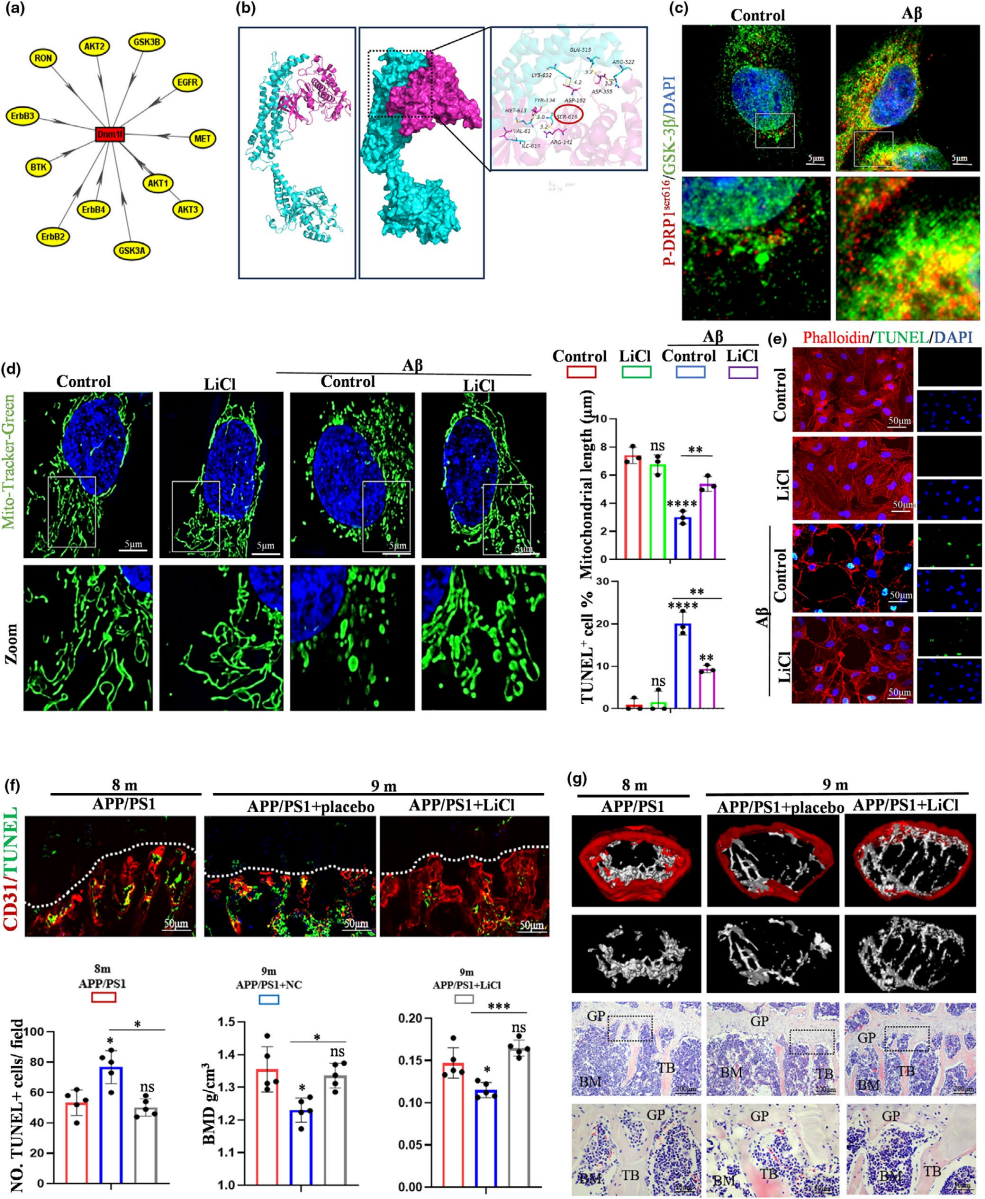
GSK‐3β was found as a key regulatory target of Aβ‐induced excessive mitochondrial fission and bone loss. (a) Predicted phosphorylated kinase of DRP1 by iGPS1.0 software. (b) The binding mode of the complex GSK3β with Drp1. The backbone of protein was rendered in tube and colored in red (GSK3β) and cyan (Drp1). GSK3β and Drp1 protein is rendered by the surface. The detail binding mode of GSK3B with Drp1. (c) Immunofluorescence of GSK‐3β; p‐Drp1^S616^; DAPI. Scale bars: 5 μm. (d) The Mito Tracker staining to assess the role of GSK‐3β inhibitor, LiCl, rescues the length of mitochondria affected by Aβ. Scale bars: 5 μm. (e) TUNEL staining shows the apoptosis of ECs and the quantitative analysis. Scale bar: 50 μm. (f) TUNEL staining to assess the role of LiCl in the apoptosis of ECs in vivo. Scale bars: 50 μm. (g) The assessment of bone mass by μCT and HE staining and quantitative parameters. Scale bar: 200, 50 μm. BMD, Bone mineral density, BV/TV, Bone Volume per Trabecular Volume. Values are presented as mean ± SEM. One‐way ANOVA with Bonferroni's multiple comparisons test was performed in d, e, f, g. (****p < 0.0001, ***p < 0.001, **p < 0.01, *p < 0.05).

It was shown that Drp1 is a critical regulator of mitochondrial fission by elevating GTPase hydrolytic activity (Jin et al., 2021). The post‐translational modifications of Drp1, such as sumoylation, S‐nitroylation, ubiquitination and phosphorylation, were assumed to be tightly associated with its regulation. Preferentially, the phosphorylation of Drp1 at ser^616^ was reported to result in the translocation of Drp1 from cytosol to the mitochondrial outer membrane. Hence, the iGPS 1.0 software was utilized to predict Drp1 phosphorylated kinase, and 12 phosphorylated kinase was found to be have relationships with Drp1 (Figure 6a). Considered the activity of GSK‐3β in the development of AD, it was speculated that Aβ‐induced excessive mitochondrial fission might be caused by the enhanced activity of GSK‐3β and the phosphorylation of Drp1 at ser^616^. Docking analysis found GSK‐3β interacted with Drp1 through hydrogen bonds at ser^616^ (Figure 6b). Next, co‐IF staining of p‐Drp1 and GSK‐3β confirmed that the treatment of Aβ promoted the co‐location of GSK‐3β and p‐Drp1 (Figure 6c). These results suggested that GSK‐3β may be an important regulatory target of Aβ‐induced excessive mitochondrial fission. To further investigate the accuracy of the proposed hypothesis, lithium chloride (LiCl) was applied to inhibit the activity of GSK‐3β. Moreover, the mitochondrial fluorescent probe staining and TUNEL staining indicated that the inhibition of GSK‐3β significantly alleviated Aβ‐induced excessive mitochondrial fission and apoptosis of ECs (Figure 6d,e). Moreover, the vivo data also identified that LiCl ameliorated the apoptosis of type H ECs in APP/PS1 mice (Figure 6f). Finally, μCT and HE staining further validated that LiCl also ameliorated the bone loss of APP/PS1 mice, compared with placebo group (Figure 6g). In summary, GSK‐3β could be a crucial regulatory target of Aβ‐induced excessive mitochondrial fission and bone loss.

## 4 DISCUSSION

Although AD patients exhibited a remarkable deterioration in bone quality, the pathological association between AD and bone loss remains elusive. Recent advances in our comprehension of the organ‐specific vascular system indicated that the impairment of bone blood vessels, particularly type H vessels, maybe hit the point. In the current study, a novel mechanism underlying the association between AD and deteriorated bone loss was presented. Initially, a higher degree of impairment in bone blood vessels was found in APP/PS1 mice during aging, leading to a more compromising vascularized osteogenesis. In addition, type H blood vessel injury was closely linked to the apoptosis of ECs, and Aβ‐induced excessive mitochondrial fission was required for the apoptosis of ECs. Furthermore, inhibition of GSK‐3β significantly alleviated Aβ‐induced excessive mitochondrial fission and type H blood vessel injury, indicating that GSK‐3β may be an important therapeutic target for bone loss in AD patients.

It was reported that bone loss always occurs in preclinical AD, highlighting its predictive value in the assessment of AD risk (Frame et al., 2020). The results of the present study confirmed that 4‐month‐old APP/PS1 mice manifested low bone mass rather than cognition impairment compared with age‐matched WT mice. Although bone loss may predate cognitive impairment, the discovery of ‘brain‐bone connection’ confirmed that cognitive impairment plays a critical role in AD patients' bone loss, suggesting a bidirectional relationship between the progression of AD and bone loss. Recently, AD and osteoporosis have been recognized as concomitant diseases of old age, sharing common molecular pathways for disease progression, including aging and genetic susceptibility (Stapledon et al., 2021). In the present study, it was revealed that abnormal deposition of Aβ had a devastating effect on bone mass and cognitive function, aligning with the analysis of concomitant diseases.

After sequential cleavage by β‐ and γ‐secretase, Aβ is generated from amyloid precursor protein (APP). This process is defined as amyloidogenic processing pathway (Hamaguchi et al., 2021). Imbalance between Aβ production and clearance leads to the accumulation of neurotoxic Aβ oligomers. Studies have reported that Aβ exerts various effects on skeletal health by impacting bone cells (Li et al., 2014; Xia et al., 2013). The findings of the present study highlighted another target of Aβ: bone vascular cells. Healthy bone blood vessels play a crucial role in maintaining bone metabolism homeostasis, and their injury is closely linked to bone loss, particularly in type H blood vessels (Hu et al., 2021; Liu et al., 2021; Wang et al., 2022). Tissue‐specific endothelium plays an essential role in the maintenance of tissue homeostasis by providing specialized vascular niches that regulate nearby cells (Potente & Mäkinen, 2017). As organ‐specialized vascular ECs, ECs in type H blood vessels were found to promote osteogenesis by secreting multiple factors to support the differentiation of osterix^+^ osteoprogenitors to osteoblasts, including Noggin and TGF‐β (Liang et al., 2021). Additionally, in the bone growth stage, type H endothelium plays a crucial role in cartilage resorption and directional bone growth by secreting proteolytic enzyme (Romeo et al., 2019). Excessive apoptosis of type H blood vessel ECs caused by Aβ may disrupt the regulation and support provided to the osteoblast lineage, leading to compromised bone formation. These findings innovatively highlight the relationship between bone blood vessel injury and AD‐related bone loss, furnishing a novel insight of AD‐related bone loss.

For the apoptosis of type H ECs, we found Aβ‐induced excessive mitochondrial fission was required. Mitochondrial dynamics are identified to be crucial to cell's survival and death (Wu et al., 2021). Aberrant mitochondrial fission will produce excessive spherical mitochondria, characterized by disappearance of cristae membrane and occasional cristae dilation or vesiculation. Those spherical mitochondria lead to the apoptosis of ECs. Drp1, a member of the dynamin family of GTP‐binding proteins, is the primary mediator of mitochondrial fission (Rahmani et al., 2023). The results of the current study revealed that Aβ could promote the transfer of Drp1 from the cytosol to the mitochondria. It was reported that the transferred Drp1 forms helical oligomers that encase the outer mitochondrial membrane in a GTP‐dependent manner, thereby facilitating mitochondrial fission. Further exploration indicated that Aβ alters the post‐translational modifications of Drp1 to promote the transfer of Drp1 from the cytosol to the mitochondria. It is broadly accepted that phosphorylation of Drp1 has the dural effect on mitochondrial fission depending on the specific residue targeted. Although the effect of phosphorylation at Ser^637^ remained controversial, phosphorylation at Ser^616^ was reported to promote the translocation of Drp1 to the mitochondria and activation of mitochondrial fission (Liu et al., 2022). The present study indicated that Aβ could promote the phosphorylation of Drp1 at Ser616 by influencing the activity of phosphorylase kinase, GSK‐3β. As another critical molecular pathogenesis of AD, GSK‐3β‐mediated hyperphosphorylation of tau protein is a hallmark of neurodegenerative tauopathies due to the intraneuronal accumulation of neurofibrillary tangles (Sayas & Ávila, 2021). In addition to the effect on hyperphosphorylation of tau protein, GSK‐3β plays a notable function in the deposit of Aβ, suggesting the bidirectional regulatory relationship between the activation of GSK‐3β and the deposit of Aβ. Collectively, it is critical to understand the complexity of bone loss complicated with AD.

As an important virulence factor, Aβ could eventually affect all kinds of cell types in bone, indicating that bone blood vessel injury is not the only mechanism for AD‐related bone loss. Moreover, no significant Aβ accumulation was observed in preclinical AD mice (4‐month‐old APP/PS1), but they had significant bone vessel injury and bone loss, indicating there may be other mechanisms regulating bone loss of preclinical AD mice. Additionally, the vivo rescue experiments were carried out by the way of systemic administration in this study, the accurate targeted delivery vector needs to be developed in the next step.

In conclusion, our results revealed that bone blood vessels injury, especially type H blood vessels, might be a critical contributor of AD‐related bone loss. Moreover, Aβ‐induced excessive mitochondrial fission drives the apoptosis of type H ECs, resulting to the type H blood vessels injury. Additionally, GSK‐3β was found to be an important target of Aβ‐induced excessive mitochondrial fission. Finally, the findings are mainly from animal models, which need to be identified in human samples. Therefore, the AD patients' clinical data should be carried out in the future.

## AUTHOR CONTRIBUTIONS

Weidong Zhang, Tomoka Hasegawa, Hongrui Liu and Minqi Li designed the study, Hongrui Liu and Minqi Li supervised the study. Weidong Zhang and Fan Ding performed most experiments and analysis. Xing Rong and Qinghua Ren involved in data statistical analysis. Weidong Zhang, Hongrui Liu and Minqi Li verified the data, wrote the manuscript, and made revisions. All authors read and approved the final version of the manuscript.

## CONFLICT OF INTEREST STATEMENT

The authors declared that they have no conflict of interest.

## Supporting information


Figure S1.

Figure S2.

Figure S3.

## Data Availability

All data that support the findings of this study are available from the authors upon request.
