# Supplementary figures and images for: Aβ ‐induced excessive mitochondrial fission drives type H blood vessels injury to aggravate bone loss in APP/PS1 mice with Alzheimer's diseases
Source: Aging Cell. 2024 Oct 16;24(2):e14374. doi: 10.1111/acel.14374 (PMC11822656; doi:10.1111/acel.14374)

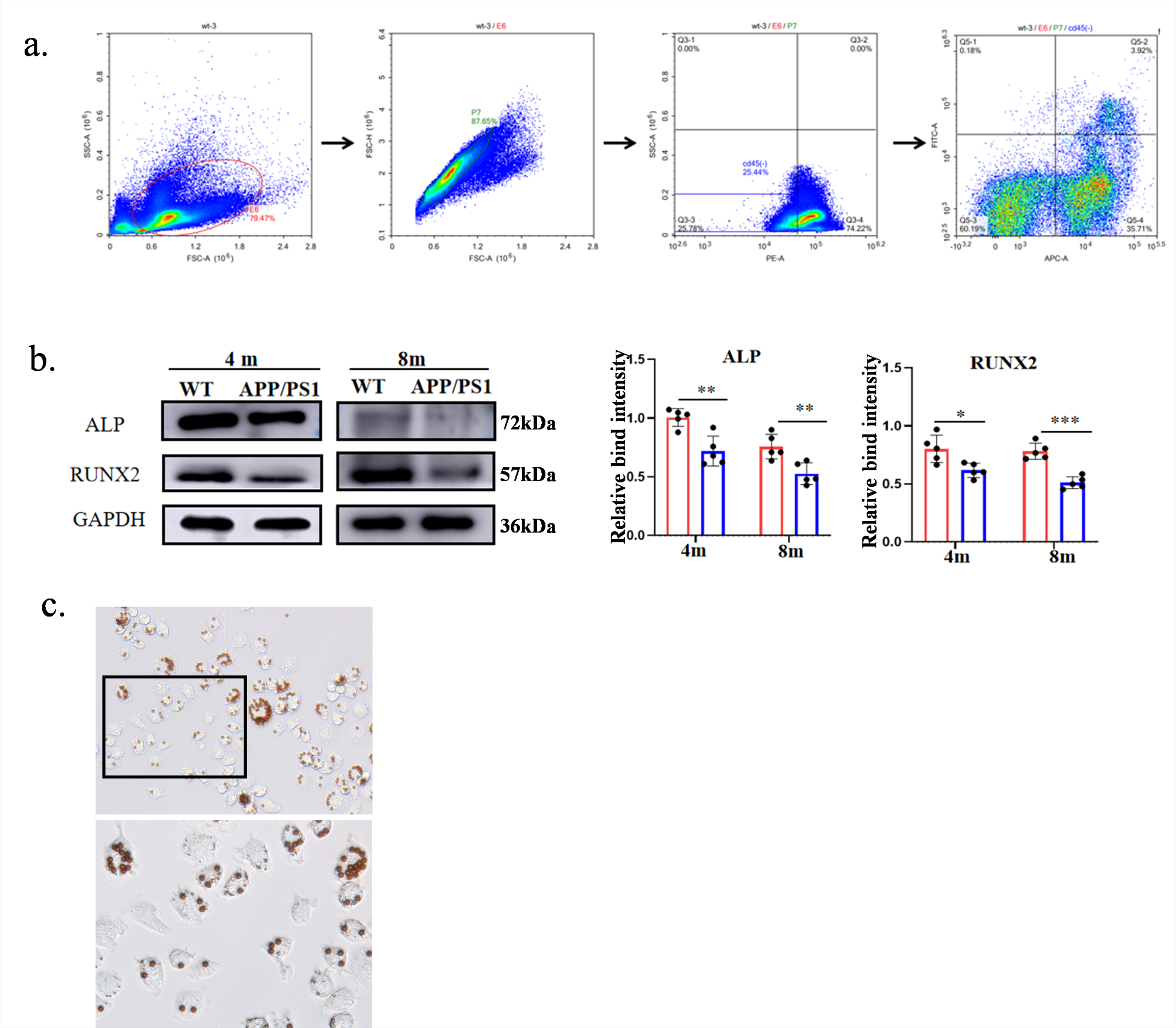

Supplement: Supplementary file 1 — Figure S1. [file ACEL-24-e14374-s001.tif]

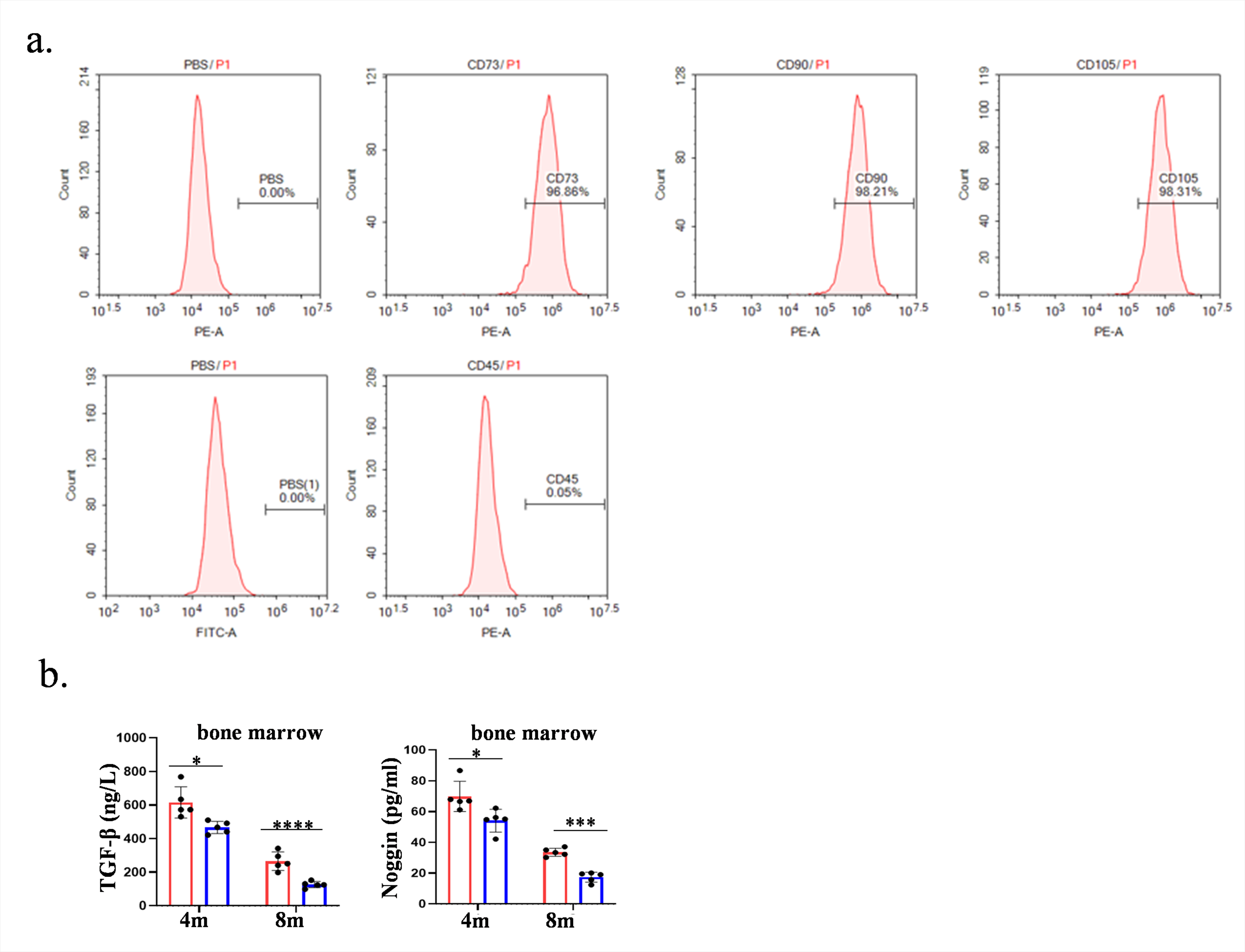

Supplement: Supplementary file 2 — Figure S2. [file ACEL-24-e14374-s002.tif]

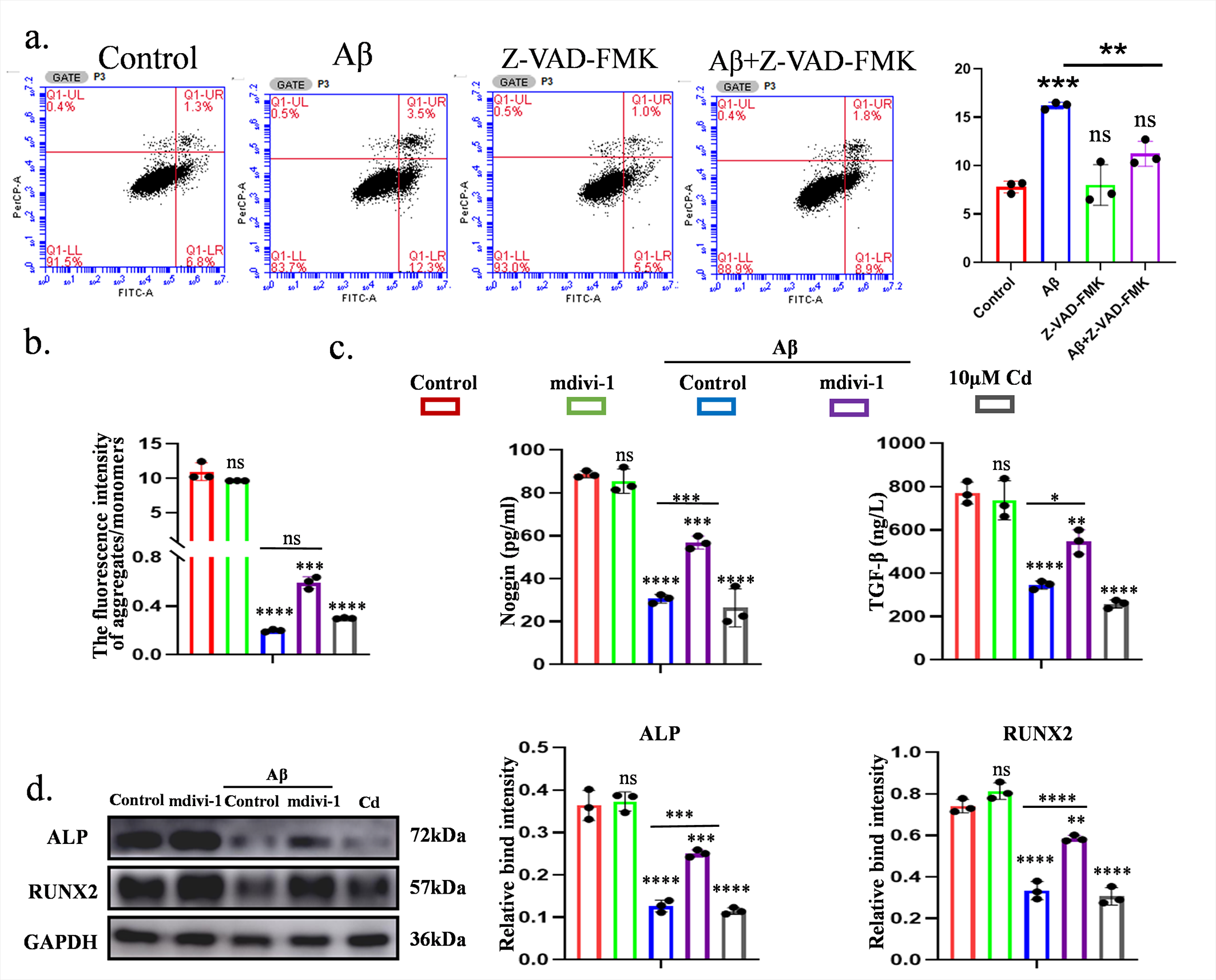

Supplement: Supplementary file 3 — Figure S3. [file ACEL-24-e14374-s003.tif]
